# *Tang Wang Ming Mu* Granule Attenuates Diabetic Retinopathy in Type 2 Diabetes Rats

**DOI:** 10.3389/fphys.2017.01065

**Published:** 2017-12-19

**Authors:** Mingxia Chen, Haibo Lv, Jiakuan Gan, Junguo Ren, Jianxun Liu

**Affiliations:** ^1^Graduate School, Beijing University of Chinese Medicine, Beijing, China; ^2^Xiyuan Hospital, China Academy of Chinese Medical Sciences, Beijing, China; ^3^Beijing Handian Pharmaceutical Co. Ltd, Beijing, China

**Keywords:** diabetic retinopathy, antihyperglycemic activity, oxidative stress, *Tang Wang Ming Mu* Granule, VEGF, JAK2/STAT3 pathway, SOCS3

## Abstract

**Aims:** This study aimed to determine the influence of *Tang Wang Ming Mu* granule (TWMM) on the diabetic retinopathy of diabetic rats.

**Methods:** Male Wistar rats were divided into seven groups: normal control, diabetes model(DM), diabetes with TWMM (3.6, 7.2, and 14.4 g/kg) treatment, the positive control treatment groups of Qi Ming granules and Calcium dobesilate capsules. All rats were treated for 8 weeks. The levels of body weight, fasting blood glucose (FBG) and glycosylated hemoglobin (HbA1c) in blood were measured to evaluate the antihyperglycemic activity of TWMM. Furthermore, malondialdehyde (MDA), intracellular adhesion molecule-1 (ICAM-1) and vascular endothelial growth factor (VEGF) in serum were measured to study effects of TWMM on oxidative stress and inflammatory in DM2 rats. VEGF, JAK/STAT signaling pathway and SOCS3 in retina was detected by immunohistochemistry.

**Results:** TWMM and the positive control drugs Qi Ming and Calcium dobesilate showed a remarkable suppression of retinal neovascularization and amelioration of retinal internal limiting membrane morphology. Moreover, TWMM significantly decreased HbA1c, MDA, ICAM-1, and VEGF levels in serum of diabetic rats. However, Qi Ming granules showed significantly reduced MDA and VEGF levels (*P* < 0.01, and *P* < 0.05, respectively), Calcium dobesilate showed significantly reduced MDA and ICAM-1levels (*P* < 0.01 and *P* < 0.05, respectively) in serum. All drug- treated DM2 rats showed significantly lower levels of VEGF, JAK2, P-JAK2, STAT3, and P-STAT3 in retina than DM group, while TWMM and Calcium dobesilate significantly increased SOCS3 in retina.

**Conclusion:** Our data suggest that the diabetic retina protective effect of TWMM might be related to antiinflammatory, antioxidative, upregulation of SOCS3 expression, inhibition of the JAK/STAT/VEGF signaling pathway.

## Introduction

A report published by the International Diabetes Federation currently indicates that diabetes mellitus has impact on at least 378 million people worldwide, and this figure is most likely to be doubled by 2035 (Yisahak et al., 2014). Diabetic retinopathy (DR) is one of the most common and serious microvascular complications of diabetes, result in visual impairment and blindness. Its incidence reaches nearly all type 1 diabetes patients and more than 60% in type 2 diabetes patients after 20 years onset of diabetes (Clark and Lee, 1995). DR is clinically characterized by retinal microvascular pathologic changes, such as capillary occlusion, hemorrhages, microaneurisms, and neovascularization, finally leads to severe vision loss and irreversible blindness (Antonetti et al., 2012; Cohen and Gardner, 2016). A number of therapeutics, such as anti-vascular endothelial growth factor (VEGF) therapy, long-acting steroids, and surgery with laser photocoagulation, has been applied clinically. However, they were used for later stage of DR. Therefore, early treatments to delay DR progression are desperately needed and have great social and economic impacts.

Although the pathogenesis remains unclear, studies have indicated that many factors, including hyperglycemia, oxidative stress, and inflammatory cytokines, contribute to DR progression. Among these factors, hyperglycemia is accepted as the first initiating factor in the development of DR, which suggests that strict glycemic control is effective in delaying DR (Chiu and Taylor, 2011). Recently, it is believed that inflammatory process plays an important role in the development of DR (Liao et al., 2017). In addition, oxidative stress is also considered as one of the crucial contributors in the pathogenesis of diabetic retinopathy, leading to structural and functional changes and accelerated loss of capillary cells in the retinal microvasculature (Jin et al., 2014). The target management for vascular complications of diabetes has been pointed out to inhibit inflammation and oxidative stress pathway.

*Tang Wang Ming Mu* Granule (TWMM), a traditional Chinese prescription, contains *Astragali radix, Leonuri herba, Buddlejae flos, Ligustri lucidi fructus, Coptidis rhizoma, Mume fructus*, as well as *Cinnamomi cortex* (Hao et al., 2016). Quantities of these ingredients are 34.8, 17.4, 13.0, 13.0, 8.7, 8.7, and 4.3% of the total weight, respectively. Presently, the herbal medicine has increasingly gained attention as a protector of diabetic vascular complications. Our previous research showed that TWMM had inhibition effect on human umbilical vein endothelial cell line EA.hy926 under hypoxic and high glucose conditions. It might be related to down-regulation of the expression of angiogenesis-related factor mRNA and proteins, such as HIF-1α, VEGFR-2, and ICAM-1 (Hao et al., 2015).

No *in vivo* study has been carried out to investigate the efficacy of TWMM to prevent or delay diabetic-induced retinal microangiopathy. The purpose of this study was to evaluate the effect of TWMM on experimental diabetic retinopathy in the rat, and to investigate the underlying mechanisms. Calcium dobesilate capsule was chosen as a positive control drug. Studies showed that it has multiple mechanisms of action which include anti-leakage action due to its inhibitory effect on VEGF production, anti-oxidant and anti-inflammatory properties. It was approved for the treatment of DR in several countries(Solàadell et al., 2017). Qiming Granule, a traditional Chinese medicine complex prescription, which is consisting of principle components extracted from Radix Astragali, Radix Puerariae, and Rdix Rehmanniae etc. It had functions of promoting the production of body fluids and benefiting qi, tonifying the Shen and nourishing the Gan, removing obstruction in the channels to improve visual acuity. And it was used in the treatment of microvascular complications of diabetes (Xiangxia et al., 2009). At the same time, we chose Qiming Granule as a positive control Chinese medicine.

## Materials and methods

### Materials

The extracts of TWMM were kindly provided by Beijing red sun Pharmaceutical Co., Ltd (Cat. No.20130829). The main peaks in HPLC profile of TWMM were identified to be astragaloside, berberine hydrochloride, linarin, specnuezhenide, and leonurine hydrochloride. The content of these constituents in TWMM was determined to be 1.19, 6.94, 4.00, 3.76, and 0.81 mg/g, respectively, by HPLC. Qi Ming granules were purchased from Zhejing wan sheng Pharmaceutical Co., Ltd (Cat. No.141023). Calcium dobesilate capsules were purchased from Beijing JingFeng Pharmaceutical Co., Ltd (Cat. No.141105).

### Animals

Male 6-week-old Wistar rats were obtained from Si bei fu experimental animal technology Co., Ltd. (Beijing, China). All animals were maintained in the laboratory with food and water *ad libitum* throughout the experiment. The use of animals in the research was in accordance with the Association for Research in Vision and Ophthalmology (ARVO) statement. All animal work was performed in accordance with the standards established by the Animal Care and Use Committee of Xiyuan Hospital and approved by the local ethics committee.

### Induction of experimental type 2 diabetic rat model (DM)

Type 2 diabetic rat model was induced as described previously (Reed et al., 2000). Rats were daily fed with high fat diet consisting of 65% regular diet, 15% pork fat, and 20% carbohydrate. After 4 weeks of dietary manipulation, the rats were rendered diabetic by intraperitoneal (IP) injection of streptozotocin (STZ, Sigma, 25 mg/kg body weight) for 2 days. One week after STZ injection, rats with blood glucose levels consistently above 16.7 mmol/L were considered diabetic. The normal control group (*n* = 10) was given regular diet for 4 weeks followed by an IP injection of solvent. After the injection, rats in the DM group continued to be fed with high fat diet and rats in the normal control group continued to be fed with regular diet for another 4 weeks. Then, we randomly divided diabetic rats into six groups (*n* = 10 in each group): (1) DM group, diabetic rats were administered with distilled water (10 mL/kg); (2) DM+L-TWMM group, diabetic rats were administered with low dose of TWMM (ig, 3.6 g/kg); (3) DM+M-TWMM group, diabetic rats were administered with middle dose of TWMM (ig, 7.2 g/kg); (4) DM+H-TWMM group, diabetic rats were administered with high dose of TWMM (ig, 14.4 g/kg); (5) DM+QM group, diabetic rats were administered with Qi Ming granules (ig, 1.4 g/kg); (6) DM+CD group, diabetic rats were administered with Calcium dobesilate capsules (ig, 150 mg/kg). The rats in the normal control group were administered with distilled water (10 mL/kg). All rats were treated for 8 weeks.

The body weight and blood glucose levels were recorded carefully every week. At the end of the experiment, hemoglobin A1c (HbA1c) values were determined using an In2It analyzer (Bio-Rad, Munich, Germany). Rats were anesthetized by 10% chloral hydrate, the blood samples were taken from the abdominal aorta, and the eyes were removed immediately.

### Determination of metabolic parameters

Individual blood sample was withdrawn from the abdominal aorta without anticoagulant for separating serum and placed into aliquots for analyses. Serum level of MDA was measured by commercial kits from Jiancheng Bioengineering Institute (Nanjing, Jiangsu Province, China, Cat. No. 20150909). Commercial enzyme-linked immunosorbent assay (ELISA) Kits for determining serum levels of sICAM-1 (Cat. No. 238951031) and VEGF (Cat. No. 238350514) were purchased from MultiSciences (Lianke) Biotech Co., Ltd. (Hangzhou, China). All experimental assays were carried out according to the manufacturers' instructions; all samples were analyzed in triplicate.

### Retinal capillary morphology

Retinal digest stretched preparation was used to observe retinal capillary morphology, and to count the numbers of pericyte (PC/mm^2^ capillary area) and vascular endothelial cells (EC/mm^2^ retinal area), according to published methods (Dietrich and Hammes, 2012).

### Histological studies of retina

Eyes were removed from rats and fixed with 4% paraformaldehyde in phosphate buffer saline. The paraffin sections (4 μm) were then stained with hematoxylin and eosin (HE) for histological evaluation. Pathological pictures of retinas were taken at 400 × under an optical microscope (Olympus BX51, Japan), respectively. Retinal injury was evaluated by changes in retinal internal limiting membrane (ILM) of structure and neovascularization.

### Transmission electron microscopy (TEM)

The eyes were enucleated, opened at the equator, fixed in 4% glutaraldehyde solution overnight at 4°C, and then postfixed in 2% osmium tetroxide. They were then dehydrated in ethanol series, and embedded in epoxy resin and processed as described previously (Jin et al., 2014). Electron micrographs of the retinal capillaries were captured at 5,000 × magnification with a transmission electron microscopy (Hitachi H−7500, Japan).

### Immunohistochemistry

Immunohistochemistry was performed as previously described (Jin et al., 2014). Paraffin-embedded tissue block was subjected to serial 5 μm sections. The slices were subjected to antigen retrieval with citric acid buffer and then were incubated with diluted primary antibodies: VEGF antibody (Abcam, Cambridge, UK) at a dilution of 1:100, JAK2, P-JAK2, STAT3, P-STAT3, and SOCS3 antibody (Cell Signaling Technology, Danfoss, USA) at a dilution of 1:100 overnight at 4°C. After washing in PBS (pH 7.4) 3 times for 3min, the sections were incubated with secondary antibody (PV-6000) for 30 min at room temperature. Subsequently, the slides were washed again in PBS and incubated with 0.01% 3,3-diaminobenzidine tetrahydrochloride (DAB) for approximately 1 min. Sections were then washed thoroughly in PBS 3 times for 5 min each, counterstained in haematoxylin for 20s, dehydrated in absolute alcohol, cleared in xylene, and mounted in synthetic resin for microscopic examination. The digital images of the sample were observed by a digital camera (Olympus BX51, Japan). The positive area and optical density (OD) of positive cells were obtained to test with mean optical density with image J (v1.46r).

### Statistical analysis

All data were presented as mean ± standard error of the mean. Differences between groups were analyzed by Statistical Package for the Social Sciences version 17.0 (SPSS 17.0). Results were analyzed by one-way ANOVA, followed by a LSD *post hoc* multiple comparisons. A value of *P* < 0.05 was considered statistically significant.

## Results

### Effects of TWMM on metabolic parameters

**Figure 1:**
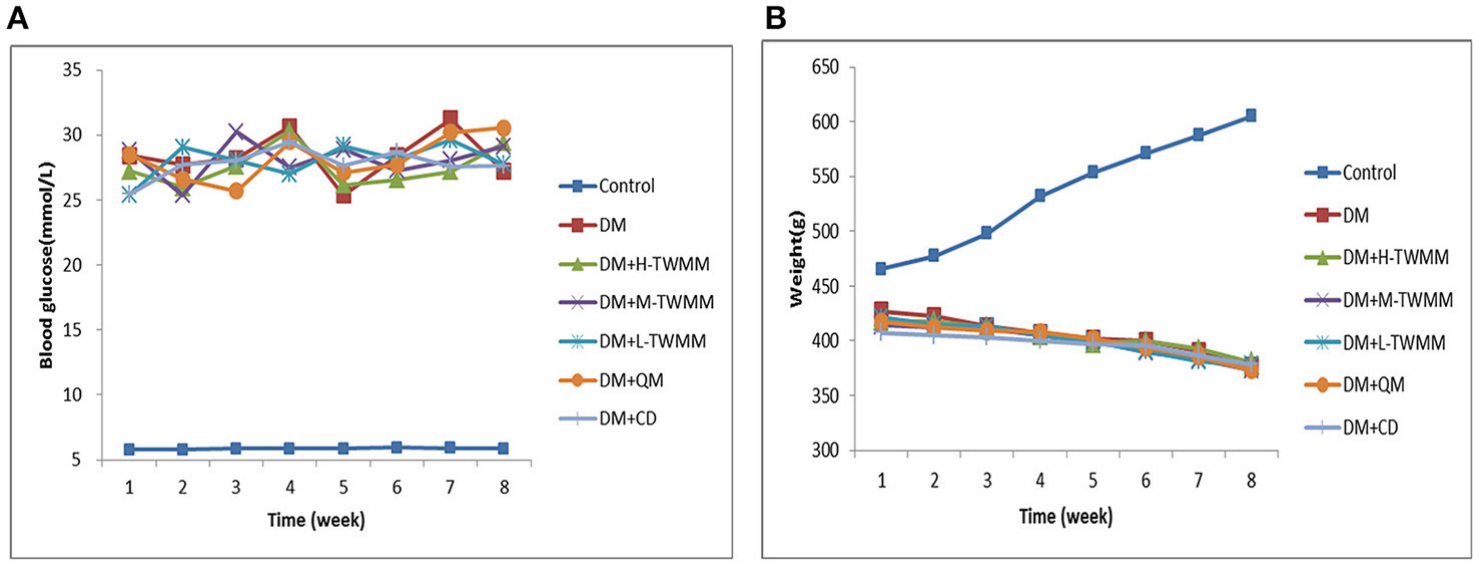
Effects of TWMM on blood glucose and weight levels in type 2 diabetic rats. Control group and model group were treated with the same volume of vehicle. Blood glucose **(A)** and weight **(B)** levels were recorded every week throughout the study. DM+H-TWMM group was treated with 14.4 g/kg TWMM. DM+M-TWMM group was treated with 7.2 g/kg TWMM. DM+L-TWMM group was treated with 3.6 g/kg TWMM. DM+QM group was treated with 1.4 g/kg Qi Ming granules. DM+CD group was treated with 150 mg/kg Calcium dobesilate capsules. Data are presented as mean. *n* = 8.

**Figure 2:**
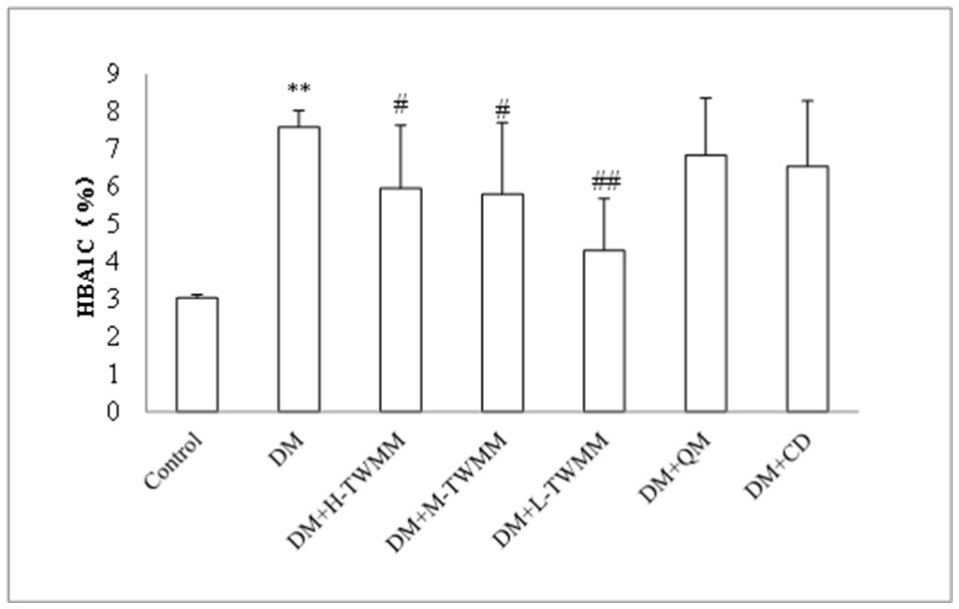
Effect of TWMM on hemoglobin A1c (HbA1c) of type 2 diabetic rats. Data are presented as mean ± SD. *n* = 8. ^**^Indicate significant differences compared to the Control group at ^**^P < 0.01. ^#,##^Indicate significant differences compared to the DM group at ^#^*P* < 0.05 and ^##^*P* < 0.01.

As shown in Figure 1A, the blood glucose levels of diabetic animals (28.41 ± 3.66 mmol/L) were significantly higher when compared with the control animals (5.78 ± 0.27 mmol/L). Eight weeks after supplementation of TWMM, although the blood glucose level of DM+TWMM groups were mildly different from that of DM rats, it showed no differences compared with the blood glucose level of DM group (Figure 1A). As shown in Figure 1B, body weights of diabetic rats were less than normal rats from week 1 to 8 (*P* < 0.01). At the end of study, there were no differences on body weight among diabetic groups. HbA1c levels of DM rats (7.59 ± 0.44%) were significantly higher than normal rats (3.03 ± 0.09%) (*P* < 0.01), confirming the impaired glucose metabolism in diabetes. The HbA1c values of DM+L-TWMM, DM+M-TWMM, and DM+H-TWMM groups revealed a significant decrease by 43.3, 23.5, and 21.5% when compared with that of DM animals, respectively (Figure 2). Compared with DM group, there were no differences on the HbA1c levels among Qi Ming and Calcium dobesilate groups (*P* > 0.05).

### Effects of TWMM on biochemical parameters of DM rats

**Figure 3:**
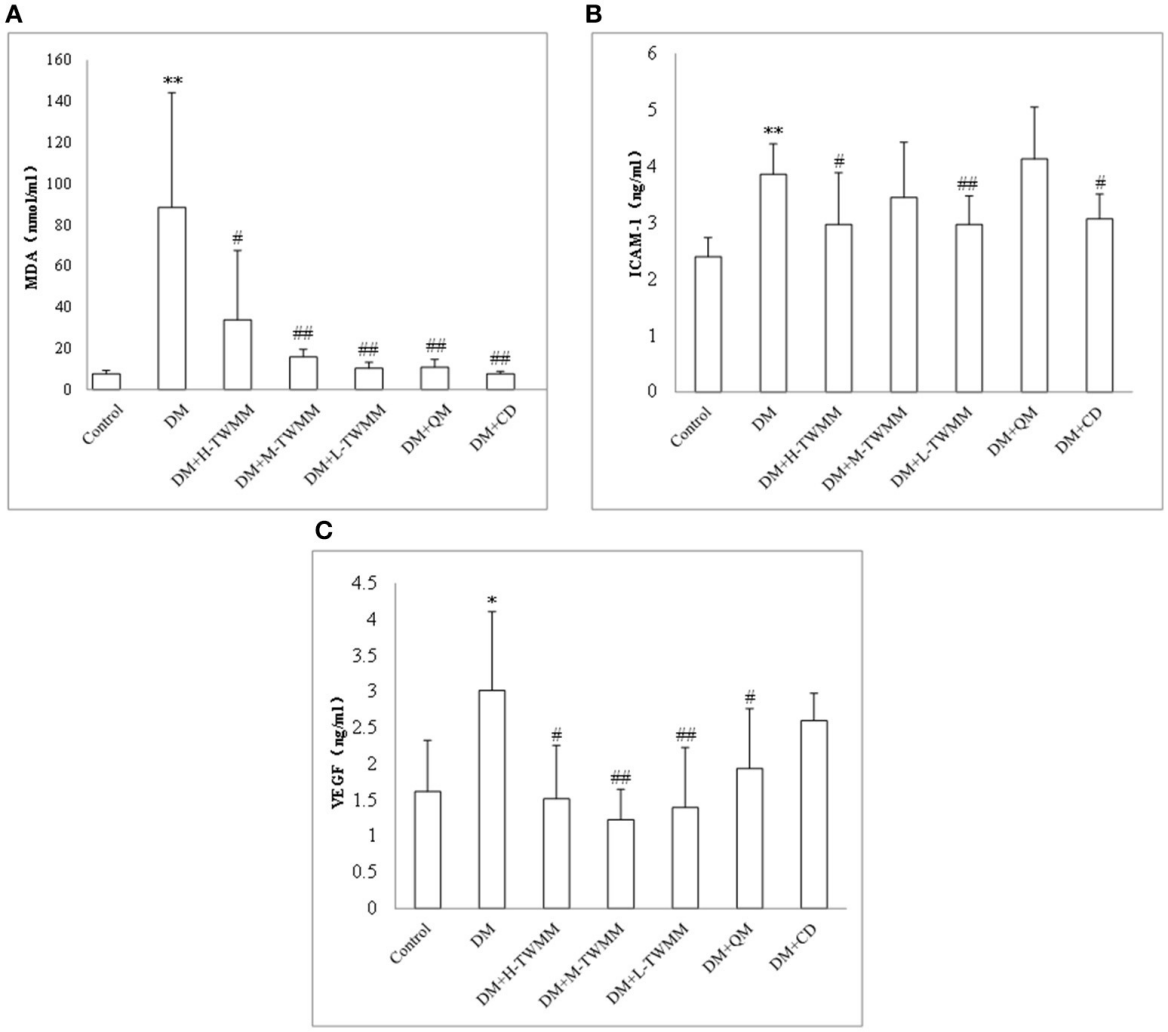
Effect of TWMM on MDA **(A)**, ICAM-1 **(B)**, and VEGF **(C)** of type 2 diabetic rats. Data are presented as mean ± SD. *n* = 8. ^*^, ^**^Indicate significant differences compared to the Control group at ^*^P < 0.05 and ^**^P < 0.01. ^#,##^Indicate significant differences compared to the DM group at ^#^*P* < 0.05 and ^##^*P* < 0.01.

Changes in malondialdehyde (MDA), intercellular adhesion molecule-1 (ICAM-1) and vascular endothelial growth factor (VEGF) were investigated. It was noted that the levels of those three parameters went to the same direction (Figure 3). The DM rats exhibited greatly higher MDA, ICAM-1, and VEGF levels than those determined in retinal tissue of the normal control rats (*P* < 0.01, *P* < 0.01 and *P* < 0.05, respectively). After 8 weeks treatment, TWMM significantly reduced MDA, ICAM-1 and VEGF levels in diabetic rats (DM+L-TWMM and DM+M-TWMM, *P* < 0.01 vs. DM group; DM+H-TWMM, *P* < 0.05 vs. DM group). Qi Ming granules treatments also showed significantly reduced MDA and VEGF levels (*P* < 0.01, and *P* < 0.05, respectively). Calcium dobesilate capsules treatments showed significantly reduced MDA and ICAM-1levels (*P* < 0.01, and *P* < 0.05, respectively).

### Effect of TWMM on experimental diabetic retinopathy

**Figure 4:**
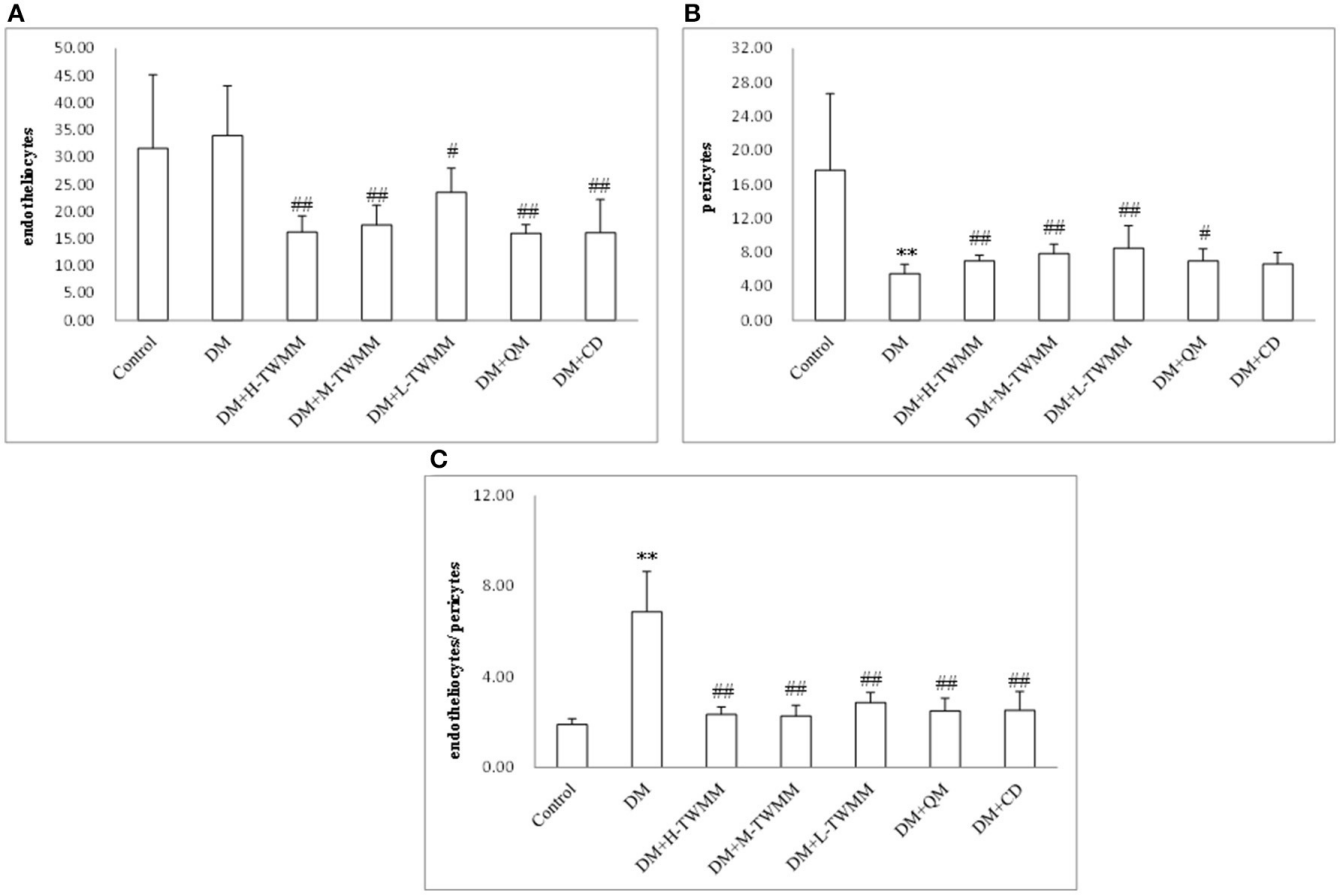
Effect of TWMM on the number of endotheliocytes **(A)**, the number of pericytes **(B)**, the ratio of endotheliocytes/ pericytes **(C)** of type 2 diabetic rats. Data are presented as mean ± SD *n* = 8. ^**^Indicate significant differences compared to the Control group at ^**^P < 0.01. ^#,##^Indicate significant differences compared to the DM group at ^#^*P* < 0.05 and ^##^*P* < 0.01.

As shown in Supplementary Figure 4A and Supplementary Image 1, quantitative analysis demonstrated that the number of endotheliocytes in model group (33.96 ± 9.15) were increased compared with that of the control group (31.63 ± 13.53), but it was no statistical meaning. All treatment groups significantly reduced the number of endotheliocytes (*P* < 0.01). As shown in Figure 4B and Supplementary Image 1, the number of pericytes in model group (5.50 ± 1.10) were decreased significantly compared with that of the control group (17.67 ± 9.00). DM+H-TWMM, DM+M-TWMM, and DM+L-TWMM groups increased the number of pericytes by 27.3, 42.9, and 54.5%, respectively (7.00 ± 0.67, 7.83 ± 1.13, 8.50 ± 2.63; *P* < 0.01). Qi Ming granules treatment also showed significantly increased the the number of pericytes by 27.3% (*P* < 0.05). Calcium dobesilate capsules treatment showed increased the the number of pericytes by 20.5%, but there were no differences with DM group (*P* > 0.05). As shown in Figure 4C and Supplementary Image 1, the ratio of endotheliocytes/ pericytes in model group (6.88 ± 1.76) significantly increased compared with that of the control group (1.90 ± 0.27). DM+H-TWMM, DM+M-TWMM, and DM+L-TWMM groups reduced the ratio of endotheliocytes/pericytes by 65.8, 66.9, and 58.3%, respectively (2.35 ± 0.33, 2.28 ± 0.47, 2.87 ± 0.44; *P* < 0.01). Qi Ming granules and Calcium dobesilate capsules treatments also showed significantly reduced the ratio of endotheliocytes/pericytes (*P* < 0.01, and *P* < 0.01, respectively).

### Effects of TWMM on retinal histology

As shown in Supplementary Image 2, the retinal layers of control group had clear structure, as well as tight and tidy cells. Moreover, retinal ILM remained smooth and integrity. However, swell ILM with protrudent capillary endothelial cells and new vessels were seen in DM group. TWMM treated groups had an obvious suppression of retinal neovascularization and reduction in swelling of ILM. There were obvious amelioration in Qi Ming granules and Calcium dobesilate capsules treatment groups.

### Effect of TWMM on ultrastructure of retinal vessels

As shown in Supplementary Image 3, the vascular endothelium was continuous and surrounded by basement membranes and pericytes in the control group. In some instances, red blood cells and platelets could be seen in the vessel, In the DM group, endothelial cells were swelled and protruded into the lumen, narrowing the vessel. The pericytes swell as well and basement membranes were uneven, thickened or split. In the TWMM treated groups, the endothelial cells and pericytes displayed minor swelling and the capillary occlusion decreased. The basement membrane thickened a little, and the vessel was in the process of repair. In the Qi Ming granules and Calcium dobesilate capsules treatment groups, the retinal ultrastructures were similar to those in the TWMM treated groups.

### Effect of TWMM on the jak/stat pathway

**Figure 5:**
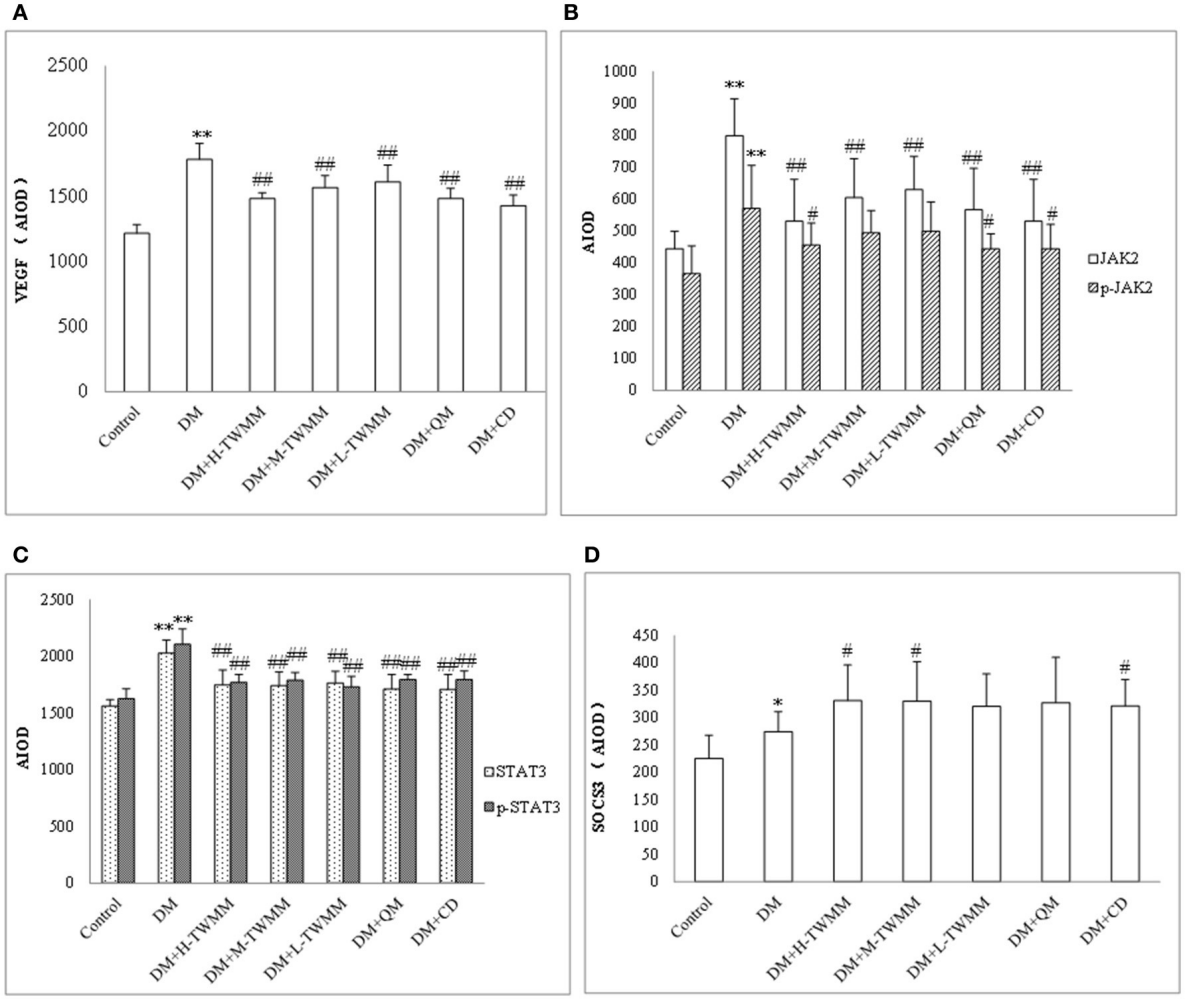
Immunohistochemistry for VEGF **(A)**, JAK **(B)**, p-JAK **(B)**, STAT3 **(C)**, p-STAT3 **(C)**, and SOCS3 **(D)** expression in the retina. Data are presented as mean ± SD *n* = 8. ^*^, ^**^Indicate significant differences compared to the Control group at ^*^P < 0.05 and ^**^P < 0.01. ^#,##^Indicate significant differences compared to the DM group at ^#^*P* < 0.05 and ^##^*P* < 0.01.

The immunohistochemistry studies demonstrated that retinal expression of JAK2 and STAT3 in the DM group were significantly higher than that in the control group (*P* < 0.01; Figure 5 and Supplementary Image 4). TWMM treatment significantly reduced retinal levels of JAK2 and STAT3 (*P* < 0.01; Figure 5B,C and Supplementary Images 4b–e). Compared with the control group, downstream of VEGF activation was increased in diabetic rats, which was suppressed by TWMM (*P* < 0.01; Figure 5A and Supplementary Image 4a). The expression of upstream protein of SOCS3 was significantly increased by TWMM (*P* < 0.05) in the middle and high dose group (Figure 5D and Supplementary Image 4f). Similarly, the expression of VEGF and phosphorylation of JAK2 and STAT3 significantly decreased in the Qi Ming granules and Calcium dobesilate capsules treatment groups compared with the DM group; whereas, SOCS3 increased in the both groups.

## Discussion

Over the last several years, multiple treatments are used in DR treatment including control hyperglycemia, laser, vitrectomy, anti-VEGF therapies, and steroids (Bandello et al., 2013). Additionally, current treatments for DR are performed in advanced stages of the disease and are associated with significant adverse effects. There has been a continuing effort to develop new drugs. Interestingly, in our study, we demonstrate for the first time that TWMM may be an effective drug for the treatment of DR.

According to the recent reports, Type 1 diabetes accounts for approximately 5% of all diabetes cases, and it has been estimated by the International Diabetes Federation that the disease affects 20–40 million persons worldwide (Tuomilehto, 2013). So, Type 2 diabetes is the majority. The prevalence of prediabetes, defined by fasting plasma glucose level, or 2-h plasma glucose, and HbA1c level, was 38% in 2011–2012, with the prevalence projected to increase to 50% by 2050 (Vinicor and Jack, 2004; Menke et al., 2015). Therefore, in this study we investigated the protective effect of the TWMM on experimental diabetic retinopathy in the type 2 diabetes rats. We use the high fat diet plus low dose of STZ injection to mimic type 2 diabetic conditions. In our study, rats with blood glucose levels consistently above 16.7 mmol/L were considered diabetic. HbA1c levels of DM2 rats were significantly higher than control group, confirming the impaired glucose metabolism in diabetes. It's very interesting that the blood glucose levels showed no reduction with those treatments, but HbA1c showed reduction. TWMM had only a minor impact on glycemia in high fat diet plus low dose of STZ injection diabetic animals, a model may be in which most of beta cells are destroyed according to the literature (Dietrich et al., 2016). The modest impact of TWMM on glycemia is reflected by the lack of differences in body weight between the diabetic groups. According to reported, there may be a discrepancy between the blood fasting plasma glucose level and HbA1c, probably caused by the undetected hyper- or hypoglycaemia over the 24-h period (Phillips and Leow, 2014). In this study, fasting plasma glucose was tested once a week. It is difficult to accurately reflect the true level of plasma glucose. However, the HbA1c reflects the average blood glucose level in the previous 2 or 3 months. So they're not consistent.

DR could be divided into non-proliferative (NPDR) or proliferative (PDR). NPDR exhibits capillary non-perfusion, cotton-wool spots, microaneurysms, dot and blot hemorrhages due to microvascular damage and pericyte loss. In PDR, neovascularization results in vitreous and retinal hemorrhages, which can lead to retinal detachment. Induction of DR by STZ, BRB breakdown could be observed 2 weeks after induction, ONL thinning beginning 4 weeks after induction, decreased numbers of both endothelial cells and pericytes, increased a cellular capillaries, after 8 weeks, and furthermore basement membrane thickening after 1 year (Olivares et al., 2017). In our study, it last 12 weeks after intraperitoneal injection of STZ. At this point, it is in the stage of non-proliferative diabetic retinopathy. Hematoxylin-eosin staining results showed that new vessels and swell ILM with protrudent capillary endothelial cells were seen in DM group. Furthermore, TEM results showed that the endothelial cells in DM group were swelled and protruded into the lumen, in addition narrowing the vessel. The pericytes swell as well and basement membranes were uneven, thickened or split. All these prove the success of the DR model in this study.

It is well recognized that hyperglycemia bring about changes in various biochemical pathways such as polyol pathway, nonenzymatic glycation end products, hexosamine pathway and diacylglycerol-PKC activation pathways. These alterations produced reactive oxygen species(ROS) which have been proposed to one of the major causes of hyperglycemia induced endothelial dysfunction (Nishikawa et al., 2000). Oxidative stress thereafter results in an increase in thrombotic tendency and a reduction in prostacyclin stimulating factors in diabetics, which may contribute to DR. In diabetes, there is an increase in free radicals production which in turn promotes lipid peroxidation. MDA is formed as an end product of lipid peroxidation, controlled by the antioxidants system under normal condition (Bhatia et al., 2003).

ICAM-1 is induced by NF-κ activation mediated by proinflmmatory cytokines, playing an important role in regulating retinal leukocyte adhesion. In addition, ICAM-1 has a close relationship with the increase of leukostasis and further breakdown of BRB during the course of DR (Kamiuchi et al., 2002).

Glycemic dysregulation, low grade inflammation and oxidative stress increases the expression of VEGF, which results in vascular leakage, macular edema as well as neovascularization in the retina (Nicholson and Schachat, 2010). In addition, VEGF provokes angiogenesis and stimulates growth and differentiation of vascular endothelial cells (Awata et al., 2002; Witmer et al., 2003). High levels of VEGF in serum and tissue play an important role in initiation and progression of retinal changes in diabetes patients (Qaum et al., 2001). Vascular alterations in the early stage of DR include acellular capillaries, microaneurysms, and thickening of vascular basement membrane (McArthur et al., 2011). These alterations occur in the early stage of DR. In this stage, vision loss is mainly caused by macular edema as the consequence of increased BRB permeability. In the present study, the increases in MDA, ICAM-1and VEGF levels were observed in retinal tissues of DM2 rats. The results suggested an obvious inflammation and BRB lesion in diabetic rats. We also have shown that the levels of MDA, ICAM-1 and VEGF in DM2 rats' retinas were significantly decreased after 8 weeks supplementation with TWMM compared with those in retinas of non-supplemented DM2 rats. The anti-inflammatory and anti-oxidative and decreases the expression of VEGF properties of TWMM seem to attribute to this protective role. Qi Ming granules treatments also showed significantly reduced MDA and VEGF levels. Calcium dobesilate capsules treatments showed significantly reduced MDA and ICAM-1levels. This might be one of the mechanisms of preventive effect on DR in DM2 rats. Compared with positive control drugs, the traditional Chinese compound preparation TWMM has component complex corresponding to multitarget therapy. However, it was difficult to observe the obvious dose-effect relationship. The mechanisms are still not well understood and need further study. In this research paper, we report for the first time a protection against diabetic microvascular damage by TWMM treatment.

When the blood glucose persistently increases, the activity of aldose reductase in pericytes of retinal capillaries increases too, resulting in enhanced level of metabolites (sorbitol and fructose) in intracellular pericytes leading to an elevation of intracellular osmotic pressure, cell swelling and metabolic disorders, eventually bringing about the loss of pericytes and damage of their primary function (autoregulation of retinal capillaries; Hammes, 2005). Without the protection of pericytes, the proliferation of endothelial cells will result in saccular outpouching of capillary walls, leading to microaneurysms with hemorrhage tendency (Ejaz et al., 2008; Pfister et al., 2008). We found that the number of retina pericytes in TWMM treated rats increased and the number of endothelial cells decreased comparing with those of STZ alone, indicating a possible mechanism that the protection of TWMM on pericytes and the inhibition of endothelial cells may attribute to its action of reducing diabetic microvascular damage. In an attempt to investigate whether the supplementation with TWMM could attenuate the retinal microvascular alterations in type 2 diabetic rats, we examined the effect of oral supplementation of TWMM on quantitative retinal morphometry. As shown in Figures 2, 4 and Supplementary Image 2, supplementation of TWMM could decrease the retinal capillary damage.

Therefore, we further looked for the mechanisms of TWMM involved in improvement of impaired retinal microvascular function in STZ combined high fat diet induced type 2 diabetes. Recent researches suggest that Janus kinase (JAK)/signal transducers and activators of transcription (STAT) signaling cascades may lead to diabetic retinopathy (Dudley et al., 2005; Al-Shabrawey et al., 2008). The JAK-STAT signaling pathway transmits information from extracellular chemical signals to the nucleus resulting in DNA transcription and expression of genes involved in proliferation, differentiation, and apoptosis (Chen et al., 2016). Recent researches showed that activation of JAK2/STAT3 plays a important role in high glucose-induced VEGF synthesis. High glucose could increase ROS production, then inducing p-JAK2/p-STAT3 and upregulating the expression of VEGF protein and mRNA in bovine retinal capillary endothelial cells (BRECs) (Zhi et al., 2010). Suppressor of cytokine signaling (SOCS) proteins form part of a classical negative feedback circuit. Transcripts encoding CIS, SOCS1, SOCS2, and SOCS3 are upregulated in response to cytokine stimulation, and the corresponding SOCS proteins inhibit cytokine-induced signaling pathways (Krebs and Hilton, 2001).

SOCS3 was normally present at low levels in resting cells, but could rapidly be induced by a variety of cytokines, hormones and some growth factors (Croker et al., 2004), as a particularly defense mechanism against cytokine-mediated apoptosis (Takase et al., 2005; Liu et al., 2008). Recent researches showed that upregulation of SOCS3 expression downregulated subsequent JAK/STAT signaling pathway, thereby reducing the destructive of beta-cell cytotoxic cytokines in diabetes (Laubner et al., 2005).

VEGF, JAK/STAT signaling pathway and SOCS3 in retina was detected by immunohistochemistry. We results showed that the expression of VEGF, JAK2, P-JAK2, STAT3, P-STAT3, and SOCS3 in retinal in the DM group were significantly higher than that in the control group. Compared with the DM group, TWMM treatment (especially the middle and high dose group) could significantly increase the levels of SOCS3 in retinal, decrease the expression of JAK2, P-JAK2, STAT3, P-STAT3, and VEGF. These suggested that the retinal protective of TWMM might be related to the upregulation of SOCS3 expression, inhibition of the JAK/STAT signaling pathway and further inhibition of VEGF expression in diabetic rat. The Qi Ming granules and Calcium dobesilate capsules treatment groups have the similar effect.

In this study, we discovered the therapeutic properties of TWMM for the prevention of diabetic microvascular disease. TWMM significantly decreased HbA1c, MDA, ICAM-1, and VEGF levels in diabetic rats. The anti-oxidative and anti-inflammatory effects of TWMM may be mechanisms contributed to preventing and delaying the procession of DR. At the same time, we observed that a correlation between STAT3-induced VEGF expression and SOCS3 induction in TWMM-treated diabetic rats' retina, suggesting that high glucose-induced activation of STAT3 and VEGF expression are under negative feedback regulation by SOCS3.

These findings contribute to a significantly better understanding of the beneficial effects of TWMM with regard to diabetes, and can thus serve as the basis for the further therapeutic development of TWMM in treating DR in future work.

## Author contributions

MC, JR, and JL conceived and designed the protocol. MC, JR, HL, and JG performed the experiments. MC, JR, and HL analyzed the data. MC wrote the paper. All the authors reviewed and approved the submitted version of the paper.

### Conflict of interest statement

Author MC was employed by company Beijing Handian Pharmaceutical Co. Ltd. The other authors declare that the research was conducted in the absence of any commercial or financial relationships that could be construed as a potential conflict of interest.
