# Supplementary figures and images for: Tang Wang Ming Mu Granule Attenuates Diabetic Retinopathy in Type 2 Diabetes Rats
Source: Front Physiol. 2017 Dec 19;8:1065. doi: 10.3389/fphys.2017.01065 (PMC5742249; doi:10.3389/fphys.2017.01065)

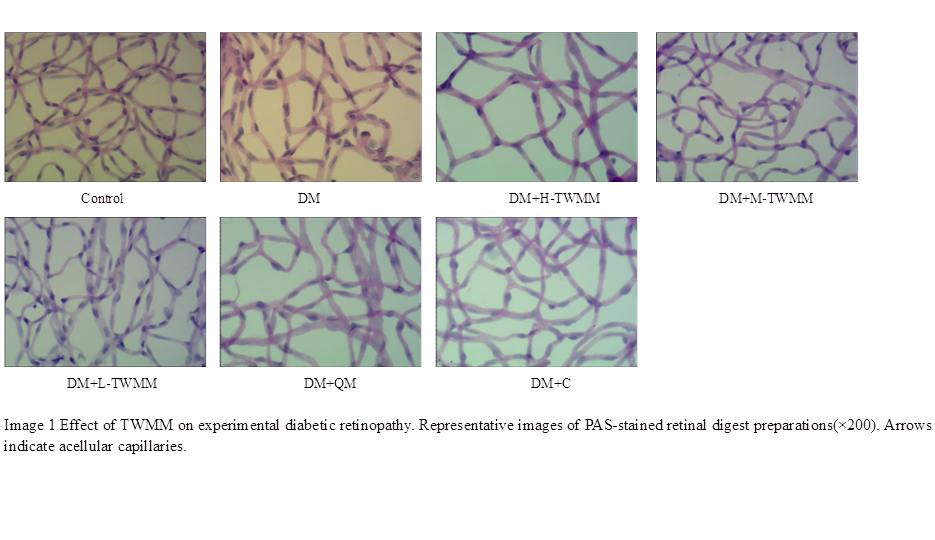

Supplement: Supplementary file 1 [file Image1.TIF]

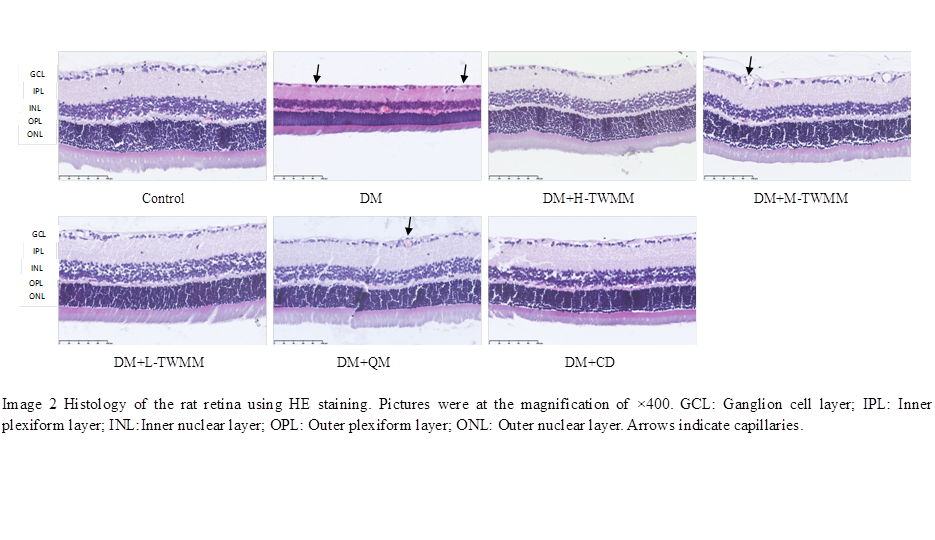

Supplement: Supplementary file 2 [file Image2.TIF]

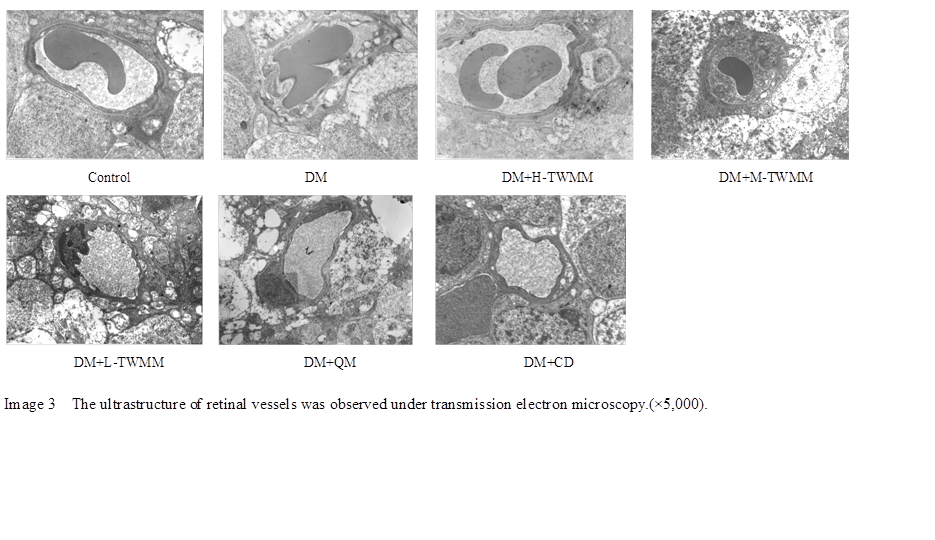

Supplement: Supplementary file 3 [file Image3.TIF]
