# Supplementary material for: Tang Wang Ming Mu Granule Attenuates Diabetic Retinopathy in Type 2 Diabetes Rats
Source: Front Physiol. 2017 Dec 19;8:1065. doi: 10.3389/fphys.2017.01065 (PMC5742249; doi:10.3389/fphys.2017.01065)
Supplement: Supplementary file 4 [file Image4.PDF]

(a)

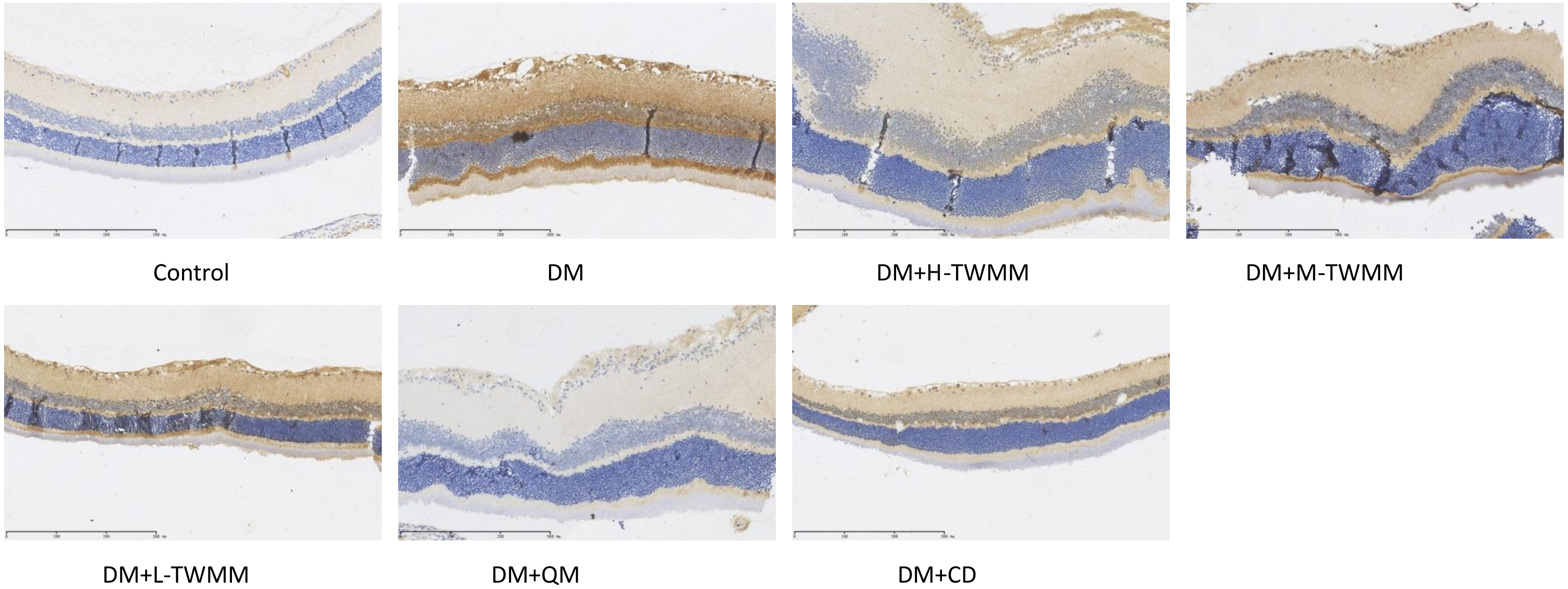

(b)

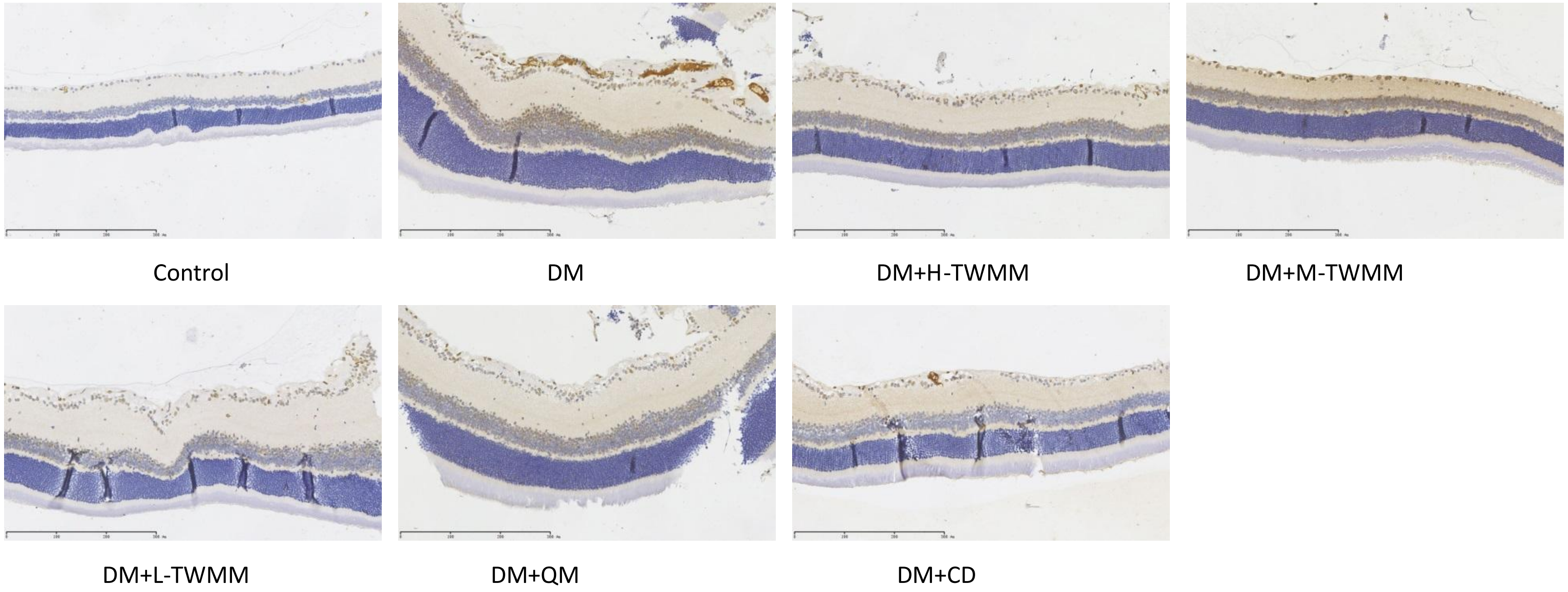

(c)

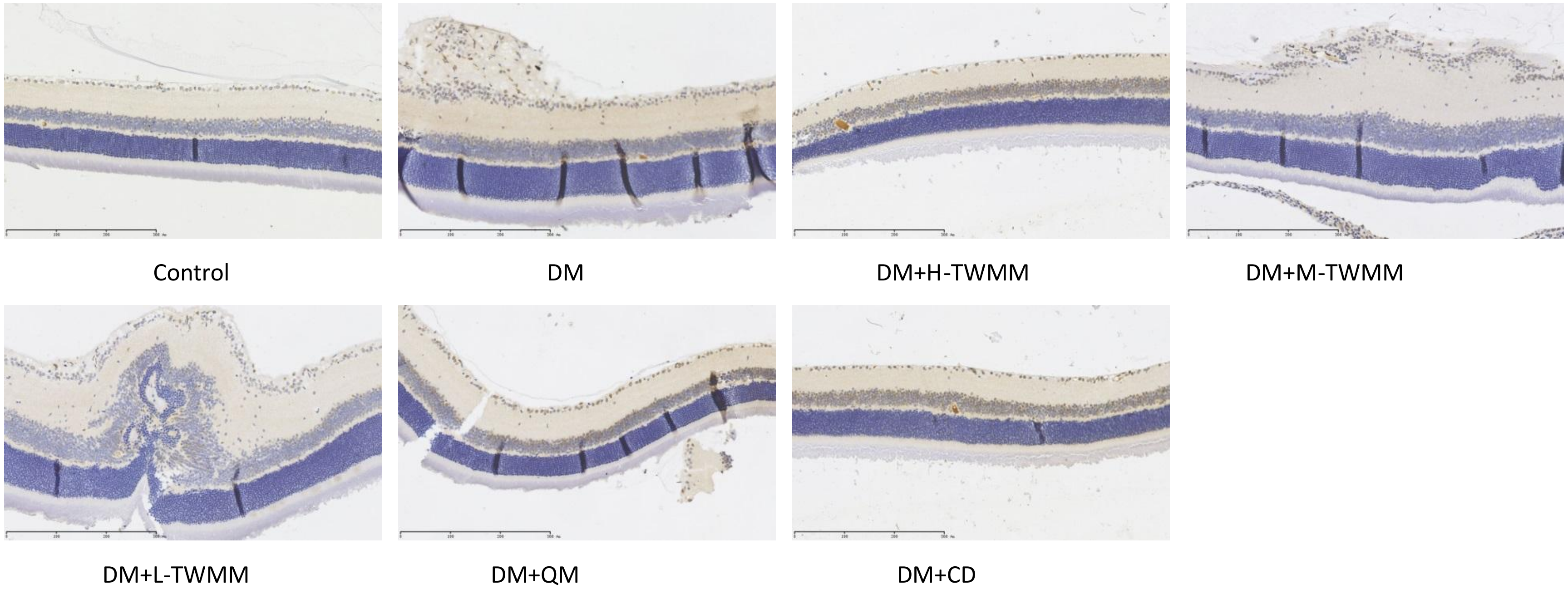

(d)

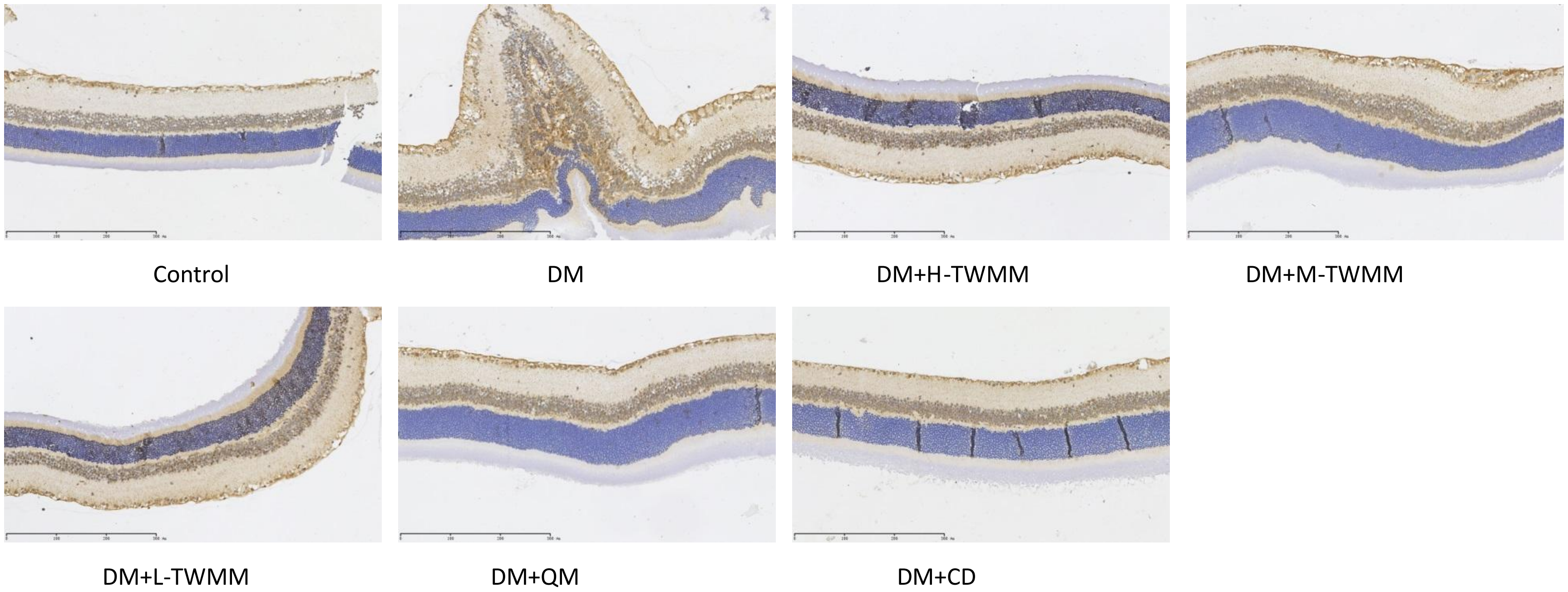

(e)

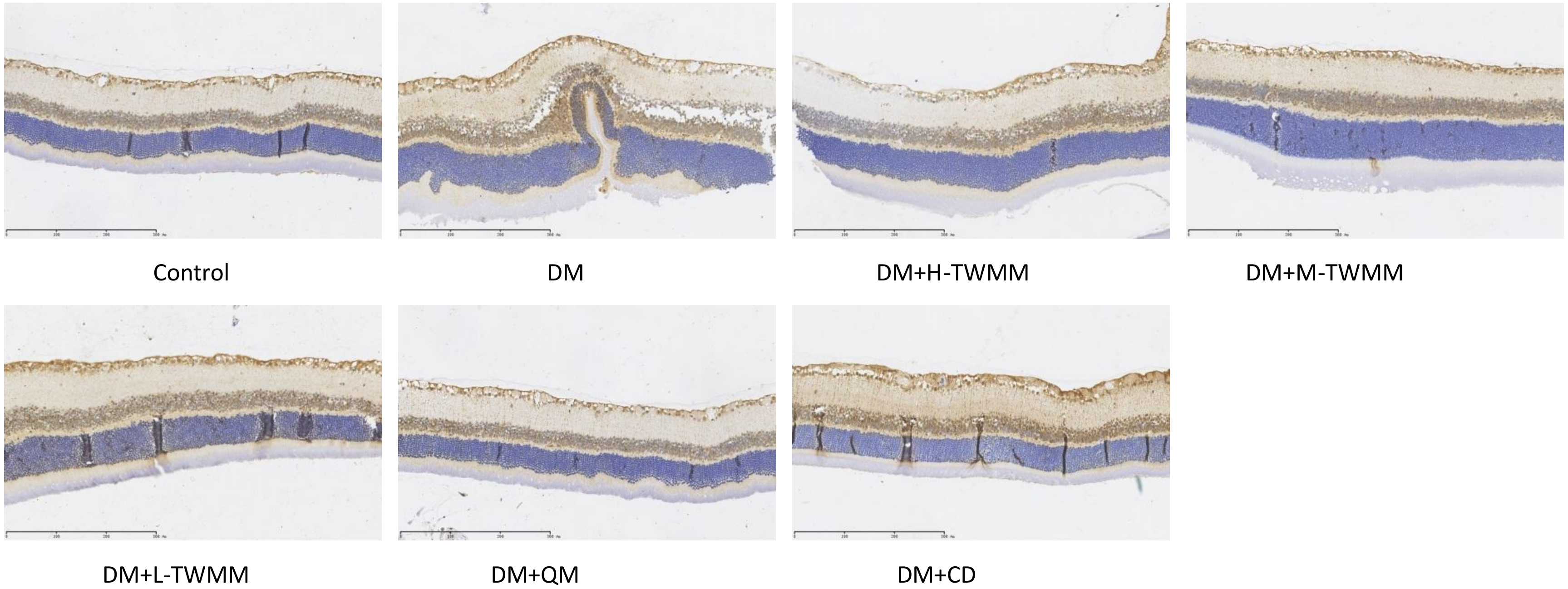

(f)

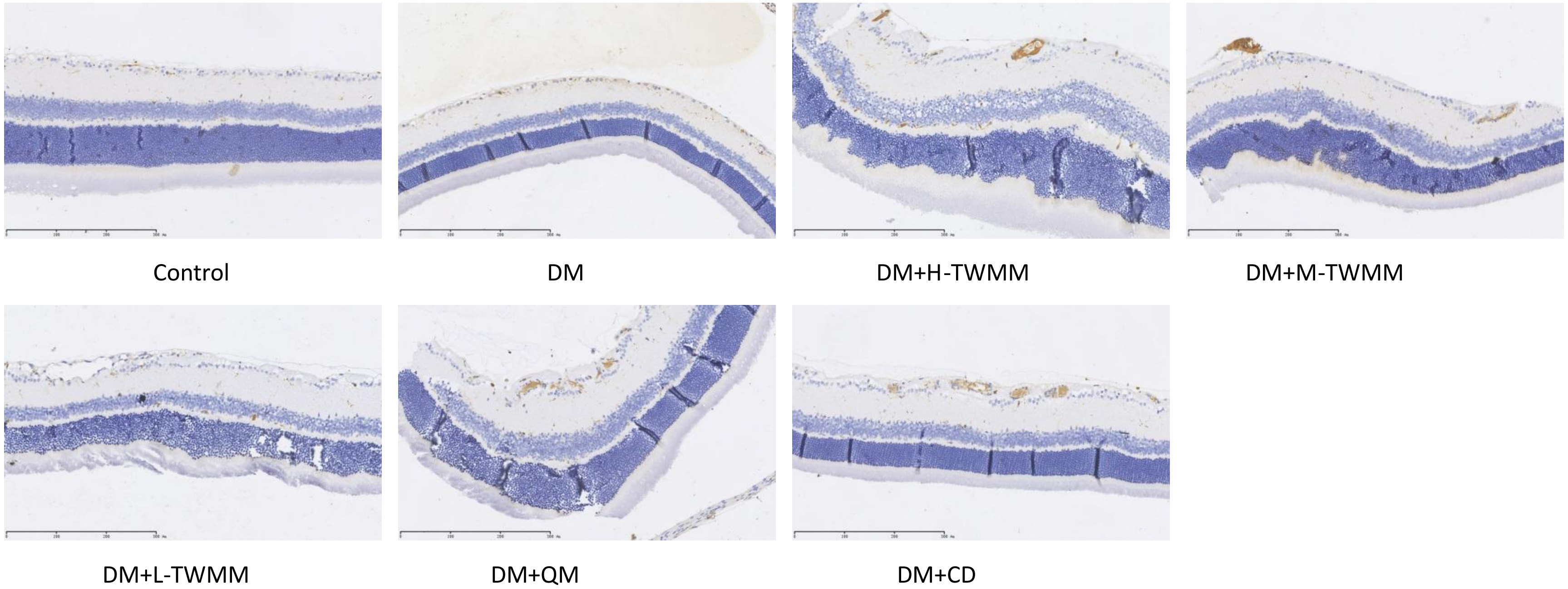

Image 4 Immunohistochemistry for the expression of VEGF (a), JAK(b), P-JAK(c), STAT3(d), P-STAT3(e) and SOCS3(f) in diabetic rat retina at the end of the treatment. Pictures were at the magnification of ×400. Control group and model group were treated with the same volume of vehicle. DM+H-TWMM group was treated with 14.4 g/kg TWMM. DM+M-TWMM group was treated with 7.2 g/kg TWMM. DM+L-TWMM group was treated with 3.6 g/kg TWMM. DM+QM group was treated with 1.4 g/kg Qi Ming granules. DM+CD group was treated with 150 mg/kg Calcium dobesilate capsules. Data are presented as mean±SD. n=8.
